# Patient Perceptions of Climate Change Impacts on Atopic Dermatitis: Cross-Sectional Survey Study

**DOI:** 10.2196/80679

**Published:** 2026-01-20

**Authors:** Gunnar Mattson, Sarah Coates, Amanda R Twigg

**Affiliations:** 1 School of Medicine, University of California, San Francisco, San Francisco, CA, United States; 2 Department of Dermatology, University of California, San Francisco, 1701 Divisadero Street, Third Floor, San Francisco, CA, 94115, United States, 1 415-353-7800

**Keywords:** atopic dermatitis, eczema, climate change dermatology, climate change, environmental health, climate health impacts, patient experience, survey study, health communication, patient education

## Abstract

This cross-sectional survey study (63.5% response rate) characterized how patients with atopic dermatitis (AD) perceive and experience the effects of climate change on their AD. Most participants reported that environmental factors such as heat and air pollution worsened their AD and expressed a desire for climate-health education, yet few had discussed these concerns with their dermatologist. These findings reveal a gap in patient-centered dermatologic care and support the development of tools to integrate environmental health into atopic dermatitis management.

## Introduction

 Climate change is recognized as the foremost global health threat of the 21st century [1]. Environmental shifts (rising temperatures, air pollution, and extreme weather) can impair the skin barrier, alter the microbiome, and induce inflammation, increasing the prevalence and severity of atopic dermatitis (AD) among other skin conditions [2,3]. Among dermatologists, 79.6% agree it affects their patients [4]. Yet, few routinely discuss this with patients, and limited research explores how patients perceive and experience these impacts. To address these gaps, this cross-sectional study surveyed patients with AD to assess how they perceive climate change’s impact on their condition and whether these concerns are addressed in dermatologic care.

## Methods

### Survey Instrument Development

The survey was informed by climate-health literature, dermatologic environmental impacts, and health communication frameworks (eg, message framing, perceived susceptibility, and severity from the Health Belief Model) [5]. Five UCSF (University of California, San Francisco) dermatologists reviewed the instrument for clinical relevance and clarity. Ten adult AD patients piloted it, and feedback informed wording and usability.

### Study Population & Recruitment

Eligible participants were English-speaking adults with AD seen at UCSF dermatology clinics between August 2023 and August 2024. A total of 2164 patients were identified by the electronic health record (EHR) query. To reduce selection bias, patients were contacted via EHR messaging or mailed letters to account for differences in digital health access; 326 patients expressed interest and became the study population. These patients were sent the study description and a secure Qualtrics link to the online survey.

### Statistical Analysis

Descriptive statistics using Microsoft Excel were used to summarize participant demographics and survey responses. Frequencies were calculated for categorical variables. No inferential or hypothesis testing was conducted, as the study aimed to characterize trends and patient-reported experiences rather than test associations or determine causality.

### Ethical Considerations

This study received exempt certification from the UCSF medical ethical review committee (IRB 21‐33538). All participants provided consent to participate in the study, and their responses were deidentified.

## Results

Of 326 individuals, 207 completed the survey (63.5% response rate). A majority of individuals (n=166/207, 80.2%, 95% CI 74.8%‐85.6%) reported that environmental-climate factors impact their AD, particularly extreme heat (n=157, 75.8%, 95% CI 70.0%‐81.7%) and poor air quality (n=81, 39.1%, 95% CI 32.5%‐45.8%). Commonly reported effects included increased medication use (n=168, 81.2%, 95% CI 75.8%‐86.5%), more symptomatic flares (n=167, 80.7%, 95% CI 75.3%‐86.1%), more skin affected (n=139, 67.1%; 95% CI 60.8%‐73.5%), and changes to daily behaviors (n=130, 62.8%; 95% CI 56.2%‐69.4%). Most participants (n=179, 86.5%; 95% CI 81.8%‐91.1%) expressed interest in understanding how environmental-climate factors affect their AD, yet only 76 participants (36.7%; 95% CI 30.1%‐43.3%) said their dermatologist addressed these concerns. The most valued strategies for addressing climate-health impacts included more information (n=164, 79.2%; 95% CI 73.7%‐84.8%), dedicated time during visits to plan for exposures (n=105, 50.7%; 95% CI 43.9%‐57.5%), and more in-person visits (n=101, 48.8%; 95% CI 42.0%‐55.6%). Table 1 shows participant characteristics, and Table 2 shows survey response data.

**Table 1.** Participant demographics and background information.

Demographics	Participants (N=207)
Age in years (mean, SD)	46.4 (18.6)
Sex, n (%)	
Male	75 (36.2)
Female	129 (62.3)
Nonbinary	3 (1.4)
Race/Ethnicity, n (%)	
American Indian or Alaskan Native	2 (1.0)
Asian or Asian American	82 (39.6)
Black or African American	12 (5.8)
Hispanic or Latino	12 (5.8)
Native Hawaiian or Pacific Islander	1 (0.5)
White	107 (51.7)
Other	5 (2.4)
Years living with atopic dermatitis (mean, SD)	21.6 (18.3)
Treatments used for atopic dermatitis, n (%)	
Topical steroid	193 (93.7)
Topical medication other than a steroid	145 (70.4)
Topical over the counter product (does not require a prescription)	139 (67.4)
Pill medication (eg, methotrexate, cellcept, tofacitinib, upadacitinib)	47 (22.8)
Injection medication (eg, dupilumab, tralokinumab)	94 (45.6)
Phototherapy	41 (19.9)

**Table 2.** Responses to survey questions using the 5-point Likert scale, where 1 indicates “Strongly disagree,” 2 “Somewhat disagree,” 3 “Neutral,” 4 “Somewhat agree,” and 5 “Strongly agree.” A reported mean greater than 3 indicates agreement and less than 3 indicates disagreement.

Statement, agreement ranked using the 5-point Likert scale	Score, mean (SD)
Climate and environmental factors have impacted your experience with eczema	4.2 (1.0)
The following factor has impacted your experience with eczema:	
Extreme Heat	4.2 (1.1)
Wildfires	3.3 (1.1)
Poor Air Quality	3.4 (1.1)
Drought	3.2 (1.1)
Extreme Rainfall	3.0 (1.3)
Sea Level Rise	2.4 (1.0)
Flooding	2.6 (1.1)
Climate and environmental factors’ impact on your eczema include:	
More symptomatic with exacerbations or flares	4.2 (1.0)
More skin affected	3.9 (1.2)
Need for extra appointments with healthcare team	3.1 (1.2)
Sending additional messages to dermatologist or calling their office	3.0 (1.2)
Using medication more often	4.1 (1.0)
Change to your medication	3.2 (1.3)
Change to lifestyle or daily behaviors	3.8 (1.1)
You want to know how the climate and environment impact your eczema	4.2 (1.0)
Your dermatologist has talked about how the climate and environment affect your eczema	2.9 (1.3)
This strategy would be helpful in managing changes to your eczema from the climate and environment:	
More visits in person	3.4 (1.1)
More telehealth visits	3.2 (1.1)
Time during visits to make plans for climate or environmental problems	3.5 (1.1)
More information on the topic	4.1 (0.9)
Support groups	2.9 (1.1)

## Discussion

### Principal Findings

While this study does not evaluate clinical causality, it provides novel insight into how patients perceive and experience the effects of environmental-climate factors on their AD. Most participants perceived climate-related changes in their AD and desired clinical guidance, yet few reported receiving it. These findings suggest that dermatologists should initiate brief conversations about common triggers, particularly heat and air pollution, and provide anticipatory guidance and resources. This insight underscores previously reported low self-efficacy among dermatologists in discussing climate change with patients [4]. Understanding these patient insights is vital to providing patient-centered care and forming effective partnerships with patients about their skin health. These efforts align with the American Academy of Dermatology’s commitment to “educate our patients about the effects of climate change on the health of their skin.” [6]

### Limitations and Future Direction

Limitations include a single-center design limiting generalizability, reliance on self-reported data with potential recall bias, and possible self-selection bias, as patients more affected by climate change may have been more likely to participate. Future research should validate these findings in broader populations, explore climate-health experiences in other skin conditions, and develop educational and clinical strategies to help navigate these climate-health conversations with patients. Even in short visits, dermatologists can explore patient experiences with climate change using supportive prompts (eg, “Would it be helpful to discuss how environmental factors might relate to your flares?”) to validate patient concerns and provide opportunities for personalized climate-health conversations to be continued in subsequent visits.

### Conclusions

This study highlights a disconnect between how patients with AD experience climate-related triggers and how often these concerns are addressed in clinical care. Findings underscore the need for tools and strategies to support climate-health conversations in dermatology. Integrating environmental health into AD management can enhance patient-centered care, improve outcomes, and reinforce dermatology’s role at the intersection of clinical care, public health, and patient advocacy.

## Abbreviations

AD: atopic dermatitis
EHR: electronic health record
UCSF: University of California, San Francisco
